# *EHP*’s Policy on Originality of Submission

**DOI:** 10.1289/ehp.1002204

**Published:** 2010-05

**Authors:** Hugh A. Tilson, Jane C. Schroeder

**Affiliations:** E-mail: tilsonha@niehs.nih.gov; E-mail: schroederjc@niehs.nih.gov

Most peer-review scientific journals will consider only work that has not been published elsewhere (Graf et al. 2007). Consistent with this policy, *EHP*’s Instructions to Authors states that “Contributions submitted to *EHP* must be original works of the authors(s) and must not have been previously published in print or online or simultaneously submitted to another publication.” According to the International Committee of Medical Journal Editors (ICMJE 2007), there are two principal reasons for such a policy, including

1) the potential for disagreement when two (or more) journals claim the right to publish a manuscript that has been submitted simultaneously to more than one; and 2) the possibility that two or more journals will unknowingly and unnecessarily undertake the work of peer review, edit the same manuscript, and publish the same article.

Therefore, a policy on originality of submissions helps to protect against copyright infringements, prevent the waste of valuable resources related to the review and publication process, and guard against possible double counting of a specific set of data or ideas.

Over the last two years, *EHP* has utilized an online search engine to help identify material that may have been published elsewhere. As a result, we discovered several manuscripts that drew heavily from reports or documents already published online by groups such as the World Health Organization, the U.S. Environmental Protection Agency, or state health or regulatory agencies. Based on our experience with these submissions, we have come to recognize that online publication of internal reports, dissertations, and other documents is not necessarily equivalent to prior publication in a peer-reviewed journal. Clearly, our policy concerning the originality of the submission needs to be updated.

To help clarify the conditions under which *EHP* will accept manuscripts based on materials that have been published online or in print elsewhere, we offer the following guidance. Much of the guidance is based on criteria developed by the ICMJE (2007). *EHP* has two different standards, one for commentaries and review articles, and one for original research papers. Previously published material may be included in commentaries and review articles, assuming that the original authors have given permission to reproduce the material and all copyright issues have been resolved. For original research articles, previously published schemata or illustrative figures are acceptable with the proper attribution; however, figures or tables of data reproduced exactly from previously published reports or other documents are not acceptable. Text or narrative from previously published reports or documents should also include new information. *EHP* will consider *a*) manuscripts from dissertations that have been published in their entirety by a university in partial fulfillment of a degree; *b*) manuscripts based on preliminary reports, such as an abstract or poster displayed at a professional meeting; and *c*) manuscripts based on papers presented at a scientific meeting but not published in full, or those that are being considered for publication in a proceedings or similar format. Previously published material may be included in the Supplemental Materials of the paper (peer reviewed but published online only).

As indicated in the guidance by the ICMJE (2007), it is the responsibility of the author to make a full statement to the editor concerning previous submissions of a manuscript or materials that might be considered redundant or duplicative.

*EHP* acknowledges the fact that considerable time and original thought is involved in producing guidance documents, technical reports, and position papers by various government and non-government organizations. Materials included in these documents may under certain circumstances be included in commentaries, reviews, and original research articles submitted to *EHP*. Hopefully, the guidance provided here will help authors in the preparation of their manuscripts. Of course, authors may wish to contact the Editor-in-Chief or Science Editor of the journal for additional guidance or information.

## Figures and Tables

**Figure:**
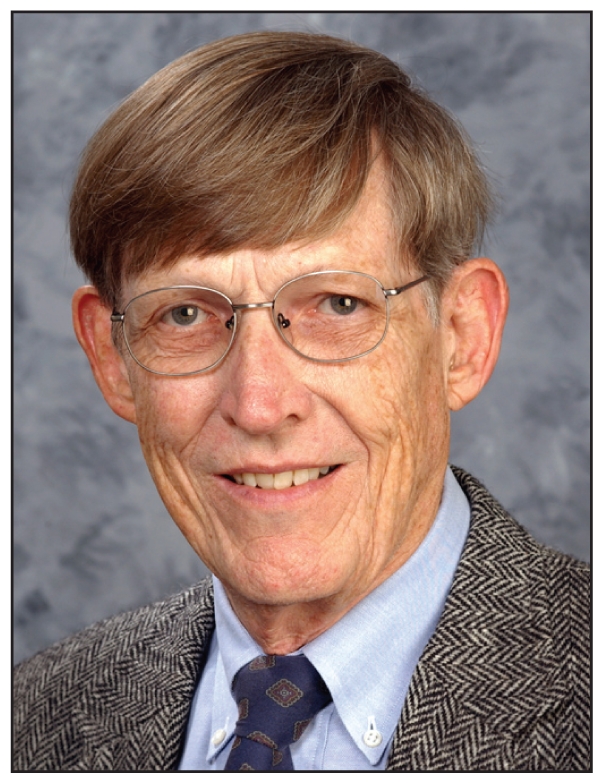
Hugh A. Tilson

**Figure:**
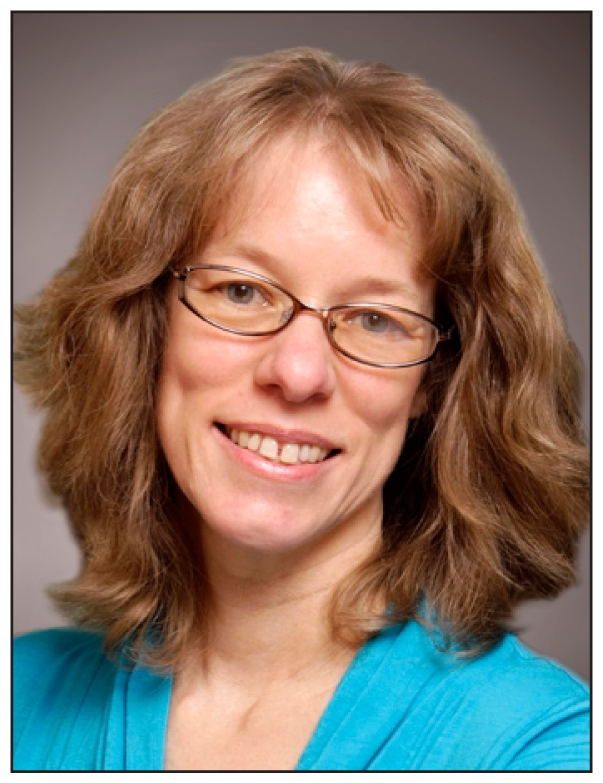
Jane C. Schroeder
